# *CENPF* Promotes Gastric Cancer Proliferation through c-Myc-Mediated GLS1 Upregulation and Glutamine Metabolism

**DOI:** 10.32604/or.2026.068508

**Published:** 2026-02-24

**Authors:** Min Dong, Zongchang Song, Xiaohui Lu, Minxue Lu, Chen Zhong

**Affiliations:** 1 Department of Oncology, No. 960 Hospital of PLA, Jinan, China; 2 Department of Oncology, Shanghai University Affiliated Mengchao Cancer Hospital, Shanghai, China; 3 Department of Oncology, The Postgraduate Training Base of Jinzhou Medical University (No. 960 Hospital of PLA), Jinan, China; 4 Department of Gastroenterology, Huzhou College Affiliated Nantaihu Hospital, Huzhou, China

**Keywords:** Centromere protein F, gastric cancer, cellular myelocytomatosis, glutaminase, glutamine metabolism reprogramming

## Abstract

**Background:**

Gastric cancer (GC) remains highly lethal, with metabolic reprogramming as a key hallmark. This study explores Centromere Protein F (CENPF)’s role in GC pathogenesis, specifically its regulation of glutamine metabolism.

**Methods:**

The Cancer Genome Atlas–Stomach Adenocarcinoma (TCGA-STAD), GSE19826, and GSE27342 datasets were analyzed by bioinformatics to identify key candidate genes in GC. The function of *CENPF* was assessed by flow cytometry, colony formation assays, and Cell Counting Kit-8 (CCK-8). RNA sequencing, metabolic profiling, chromatin immunoprecipitation (ChIP), western blot (WB), and luciferase reporter assay were employed to investigate the fundamental mechanisms.

**Results:**

*CENPF* was upregulated in GC tumor samples and had a high diagnostic potential. *CENPF* knockdown declined cell proliferation, caused G2 arrest, and promoted apoptosis in GC cells. RNA sequencing revealed that *CENPF* was involved in glutamine metabolism. *CENPF* overexpression enhanced glutamine consumption and glutamate production, while glutamine deficiency reversed *CENPF*-mediated cell survival. CENPF stabilized cellular myelocytomatosis (c-Myc) by preventing proteasomal degradation, bound to the glutaminase (GLS) promoter, promoting glutamine metabolism. Overexpression of GLS or *c-Myc* rescued the *CENPF* knockdown’s inhibitory effect on GC cell growth.

**Conclusion:**

Our findings identify a new *CENPF*/*c-Myc*/*GLS* axis that affects glutamine metabolism and cell survival in GC, implying that *CENPF* might be a novel target for the treatment of GC.

## 1 Introduction

Gastric cancer (GC) is a widely occurring cancer on a global scale, with its incidence steadily increasing [1]. Studies have indicated that the global burden of GC is expected to increase by 62% by 2040 [2]. The primary etiological factor for GC is infection with helicobacter pylori [3]. Chronic infection with this bacterium can lead to a cascade of gastric disorders, which, if left untreated, may ultimately progress to GC. In addition, dietary habits, environmental factors, and genetic predispositions have also been shown to influence the onset and progression of this malignancy significantly. Currently, the most common treatment options for GC remain surgical resection, along with chemotherapy, radiation therapy, and targeted therapies [4]. Based on its increasing incidence and multifactorial etiology, it is important to create innovative diagnostic biomarkers, therapeutic strategies, and prognostic indicators for GC.

As research into cancer treatment has advanced, targeted therapies aimed at amino acid metabolism have surfaced as a viable new direction for GC therapy [5]. These metabolic alterations are integral to the capacity of tumor cells to adapt to their environment and support their rapid growth. Glutamine is involved in key physiological processes, such as cellular metabolism, immune system function, gut health, and the regulation of the nervous system [6,7]. Common forms of amino acid metabolism reprogramming include the reprogramming of glutamine metabolism, arginine metabolism, and tryptophan metabolism, among others [8]. Glutamine is the most abundant free amino acid in plasma and participates in a variety of metabolic reactions and serves as a carbon and nitrogen donor, including the biosynthesis of nucleotides and amino acids and the replenishment of the tricarboxylic acid (TCA) cycle. Cancer cells often show increased glutamine uptake and utilization, a phenomenon known as “glutamine addiction”. This metabolic dependence has been observed in a variety of malignant tumors. Li et al. developed a risk model based on the analysis of two GC-related datasets, incorporating glutamine metabolism-related genes *MYB*, *LRFN4*, *LMNB2*, and *SLC1A5* [9,10]. This model showed a negative linkage with immune checkpoint inhibiting efficacy, revealing the function of glutamine metabolism in the progression of GC. Other studies have demonstrated that in HER2-positive GC, tumor cells encourage M2 macrophage polarization, glutamine metabolism, and angiogenesis by secreting GLS1-enriched microvesicles, which ultimately contribute to trastuzumab resistance [11]. Collectively, these findings highlight glutamine metabolism as a key mediator of tumor progression and therapeutic resistance in gastric cancer, underscoring its potential as a therapeutic intervention.

Centromere Protein F (*CENPF*) is an essential element within the G2 phase of the cell progression that encodes a protein linked to the centromere-centromere complex [12]. Numerous investigations have demonstrated that an increased level of *CENPF* is often correlated with poor prognosis in various cancers, suggesting its potential role in tumor proliferation, invasion, and metastasis. Research by Tan et al. identified that cyclin B1 (*CCNB1*), *CENPF*, and neutrophils play critical roles in diagnosing lung cancer, with high expression levels of both *CCNB1* and *CENPF* linked to poor prognosis in lung cancer patients [13]. Additionally, studies have shown that heterogeneous nuclear ribonucleoprotein R (hnRNPR) can stabilize *CCNB1* and *CENPF* mRNA expression, thereby facilitating gastric cancer progression [14]. In colon cancer cells, independent methods have confirmed changes in the phosphorylation of BET1, CENPF, and cofilin-1, as well as the significant impact of gal-4 treatment on cellular glutamine uptake [15]. In summary, these findings suggest that *CENPF* may serve as a key regulator of tumor progression and deserve further exploration in GC.

A vital variable in the progression of GC is metabolic reprogramming. Recent studies have highlighted its impact on cellular growth, survival, and therapeutic resistance [16]. Among the various metabolic pathways, glutamine metabolism has emerged as a key determinant of GC cell behavior [17]. Genes regulating amino acid metabolic pathways may shape the altered metabolic landscape in GC. However, the exact molecular mechanisms linking *CENPF* to glutamine metabolism and GC progression are not yet fully understood. In this study, we aimed to examine the role of *CENPF* in regulating glutamine metabolism. We also investigated its influence on GC cell proliferation, apoptosis, and overall tumor growth. By elucidating the interaction between *CENPF*, cellular myelocytomatosis (*c-Myc*), and glutamine metabolism, we seek to identify novel therapeutic targets. This work also provides insights into metabolic reprogramming in GC, with potential implications for the development of more effective treatments.

## 2 Materials and Methods

### 2.1 Data Collection and Analysis

We first obtained 375 Stomach adenocarcinoma (STAD) samples and 32 adjacent normal samples from the Cancer Genome Atlas (TCGA) database (https://www.cancer.gov/ccg/research/genome-sequencing/tcga) through the ASSISTANT for Clinical Bioinformatics website (https://www.aclbi.com/static/index.html#/) for this investigation. Additionally, we retrieved two relevant datasets, GSE19826 and GSE27342, from the Gene Expression Omnibus (GEO) database (https://www.ncbi.nlm.nih.gov/gds/). The GSE19826 dataset comprises 15 normal tissue samples and 12 GC tissue samples, while the GSE27342 dataset includes 80 GC tissue samples and 80 normal tissue samples.

### 2.2 Differential Expression Analysis

Differential gene expression analysis was conducted by the “Limma” package in R software (version 3.6.3). Genes in GC-related datasets (TCGA-GC, GSE19826, GSE27342) were selected based on fold change (FC) thresholds, with genes exhibiting FC > 2 considered upregulated, FC < 0.5 as downregulated, and the criterion for statistical significance is a *p* < 0.05. This approach enabled the detection of differentially expressed genes (DEGs) linked to GC progression. To visualize the results, volcano plots were generated for each dataset, with upregulated and downregulated genes highlighted in different colors.

### 2.3 Topological and Protein-Protein Interaction (PPI) Network Analysis

To analyze the relationship between DEGs across datasets, the Venn online graph tool (https://bioinformatics.psb.ugent.be/webtools/Venn/) was used to perform topological analysis on all upregulated and downregulated DEGs from the TCGA-STAD, GSE19826, and GSE27342 datasets, resulting in the identification of overlapping DEGs. The protein-protein interaction (PPI) network of overlapping DEGs was established by the Search Tool for the Retrieval of Interacting Genes (STRING, https://string-db.org/) and visualized using Cytoscape software (version 3.9.0). The Molecular Complex Detection (MCODE) and Maximum Clique Centrality (MCC) algorithms of the Cytohubba plugin were then used to recognize key genes with high centrality in the network. Intersection analysis of gene sets from MCODE and MCC was then performed to identify overlapping genes.

### 2.4 Gene Expression and Prognostic Analysis

Gene expression analysis of candidate genes in normal and tumor samples was conducted using the ASSISTANT for Clinical Bioinformatics platform (https://www.aclbi.com/static/index.html#/). Expression data from the TCGA-STAD dataset were visualized to compare gene expression between tumor and normal groups. Additionally, data processing and boxplot visualizations for the GSE19826 and GSE27342 datasets were analyzed using the Sangerbox platform (version 3.0, http://vip.sangerbox.com/home.html). In addition, the “pROC” program in R software (version 1.17.0.1) was implemented to analyze receiver operating characteristic (ROC) curves in order to assess the diagnostic value of candidate genes. The area under the curve (AUC) value of each gene was calculated in the GSE19826 and GSE27342 datasets. The sensitivity was plotted against 1-specificity, and the diagnostic accuracy of each gene was measured by the AUC value.

### 2.5 Cell Lines and Culture Conditions

**Figure A1:**
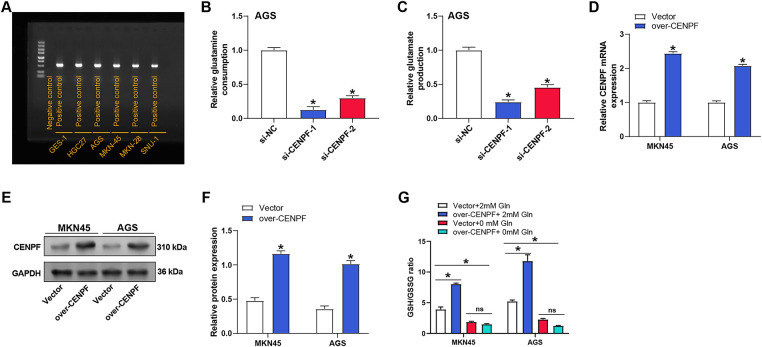
Effect of *CENPF* overexpression on glutamine consumption, glutamate production, and validation in AGS and MKN45 cells. (**A**) Mycoplasma detection results of all tested cell lines, including normal gastric epithelial cell line GES-1 and GC cell lines (HGC27, AGS, MKN45, MKN28, and SNU1), via PCR, with no specific bands observed, indicating no mycoplasma contamination. (**B**) Glutamine consumption assay showing the effect of *CENPF* knockdown on glutamine uptake in AGS cells. (**C**) Glutamate production assay demonstrating the impact of *CENPF* knockdown on glutamate generation in AGS cells. (**D)** RT-qPCR analysis of *CENPF* overexpression efficiency in MKN45 and AGS cells transfected with over-*CENPF* or a negative control vector. (**E**,**F**) WB analysis confirms *CENPF* overexpression efficiency in MKN45 and AGS cells, with quantitative results shown in the bar graphs. (**G**) RT-qPCR: Reverse transcription quantitative polymerase chain reaction. *p < 0.05 vs. si-NC or Vector or Vector + 2 mM Gln, ns: non-significant

Normal gastric epithelial cells (GES-1) and GC cell lines, such as HGC27, AGS, MKN45, MKN28, and SNU1, were obtained from Biovector (Biovector NTCC Cell Culture Collection, Inc., Beijing, China). All cell lines were authenticated by STR profiling, except for the MKN28 cell line, which has been reported as a derivative of MKN74 [18]. All cell lines tested negative for mycoplasma contamination (Fig. A1A). Cells were maintained in RPMI-1640 medium (Gibco, New York, NY, USA) supplemented with 10% fetal bovine serum (FBS, Thermo Fisher Scientific, Waltham, MA, USA) and 1% penicillin-streptomycin (Beyotime, Shanghai, China) solution. For glutamine deprivation experiments, MKN45 cells were cultivated in glutamine-free RPMI-1640 medium supplemented with 1% penicillin/streptomycin, 10% FBS, and 0 mM glutamine (Gln, MedChemExpress, Monmouth Junction, NJ, USA) for 24 h before treatment. The control group was cultured in standard RPMI-1640 medium (containing 2 mM glutamine). The cells were cultured in a humidified atmosphere containing 5% CO_2_ at 37°C. Cells were passaged when they reached 80% confluence, and the medium was replaced every two to three days.

### 2.6 Cell Transfection

AGS and MKN45 cells were planted in 6-well plates and cultivated until they reached 60%–70% confluence in order to perform gene knockdown and overexpression assays. GenePharma (Shanghai, China) produced small interfering RNAs (siRNAs) that target *GLS* (si*-GLS*) and *CENPF* (si-*CENPF*-1 and si-*CENPF*-2) as well as a negative control siRNA (si-*NC*). The pcDNA3.1 vector was used to create the CENPF overexpression plasmid (over-*CENPF*), the *c-Myc* overexpression plasmid (over-*c-Myc*), the *GLS* overexpression plasmid (over-*GLS*), and their matching empty vector controls (Vector). Following the manufacturer’s guidelines, cell transfection was carried out by the Lipofectamine 3000 reagent (Invitrogen, Carlsbad, CA, USA). Cells were taken for further testing 48 h after transfection.

### 2.7 Cell Treatment

In GLS inhibition tests, cells were treated with the GLS inhibitor CB-839 (10 μM) (MedChemExpress, Monmouth Junction, NJ, USA), which blocks glutamine metabolism in cancer cells, or an equivalent amount of DMSO (vehicle control) for 24 h. To investigate the regulatory mechanism of *CENPF* on *c-Myc*, MKN45 cells were treated differently. First, MKN45 cells transfected with si-NC or si-*CENPF*-1 were treated with MG132 (10 μM) (MedChemExpress, Monmouth Junction, NJ, USA), a specific proteasome inhibitor, for 6 h to examine protein degradation via the proteasome pathway, and second, to assess the stability of c-Myc protein, cycloheximide (CHX) chase assays were performed. After transfection with control vector or *CENPF* overexpression plasmid or si-NC or si-*CENPF*-1, cells were treated with CHX (100 μg/mL) (MedChemExpress, Monmouth Junction, NJ, USA) to inhibit protein synthesis. Cells were harvested at 0, 0.5, 1, 2, and 3 h after CHX treatment and subsequently subjected to protein analysis.

### 2.8 Reverse Transcription Quantitative Polymerase Chain Reaction (RT-qPCR)

Total RNA was isolated from cells (GES-1, HGC27, AGS, MKN45, MKN28, and SNU1) utilizing TRIzol reagent (Tiangen, Beijing, China) following the manufacturer’s procedure. RNA concentrations were quantified utilizing a NanoDrop 2000 spectrophotometer (Thermo Scientific, Waltham, MA, USA). Complementary DNA (cDNA) was synthesized by the PrimeScript RT Reagent Kit (RR047A, Takara, Kameoka, Japan). SYBR Green PCR Master Mix (RR066A, Takara, Kameoka, Japan) was employed for the RT-qPCR with an ABI 7500 Real-Time PCR System (Applied Biosystems, Foster City, CA, USA). The 2^−ΔΔCT^ technique was applied to determine the relative gene expression, with *GAPDH* serving as the internal reference [19]. The primers of RT-qPCR were as follows: *CENPF* (forward 5^′^-AAGCCTCTGTGCCGTTGAAT-3^′^ and reverse 5^′^-CAGTGAAACCACCAGCAGGA-3^′^), *c-Myc* (forward 5^′^-GGCAATGCGTTGCTGGGTTA-3^′^ and reverse 5^′^-TGATCAAGAGTCCCAGGGAGA-3^′^), *GLS* (forward 5^′^-GCTCTTAAGGCCGCCCG-3^′^ and reverse 5^′^-GATCTCCGAGGGCGAACTG-3^′^). *GAPDH* functioned as the internal control, with the primer sequences as follows: forward 5^′^-GGGTCCCAGCTTAGGTTCAT-3^′^ and reverse 5^′^-TGAGGTCAATGAAGGGGTCG-3^′^.

### 2.9 Western Blot (WB) Analysis

Cells (GES-1, HGC27, AGS, MKN45, MKN28, and SNU1) were lysed in RIPA buffer (Beyotime, China) containing protease inhibitors (Thermo Fisher Scientific, Waltham, MA, USA) for protein extraction. A BCA Protein Assay Kit (P0012, Beyotime, Shanghai, China) was applied to measure the protein concentration. SDS-PAGE was implemented to separate equal quantities of protein (30 μg), which were then transferred onto PVDF membranes (Beyotime, Shanghai, China). After blocking the membranes for an hour at room temperature with 5% non-fat milk in TBS-T, then incubated at 4°C overnight with primary antibodies against CENPF (1:1500, ab5, Abcam, Cambridge, UK), c-Myc (1:5000, Cat. No.: 67447-1-Ig, Proteintech, Wuhan, China), CDK1 (1:1000, ab265590, Abcam, Cambridge, UK), GLS (1:5000, Cat. No.: 66265-2-Ig, Proteintech, Wuhan, China), and GAPDH (1:10000, ab181602, Abcam, Cambridge, UK). The membranes were treated with HRP-conjugated secondary antibodies (goat anti-rabbit, 1:10,000, ab6721, Abcam, Cambridge, UK; goat anti-mouse, 1:10,000, ab6728, Abcam, Cambridge, UK) for one hour at room temperature following TBS-T washing. All antibodies used in this study, except for those targeting c-Myc and GLS, were purchased from Abcam (Cambridge, UK). The c-Myc and GLS antibodies were procured from Wuhan Sanying Biotechnology (Wuhan, China). Protein bands were identified using Enhanced Chemiluminescence (ECL) Western blotting Substrate (P0018AS, Beyotime, Shanghai, China) and presented with ImageJ software (*version* 1.8.0, National Institutes of Health, Bethesda, MD, USA).

### 2.10 Flow Cytometry Analysis

In order to evaluate cell apoptosis and cell cycle distribution in AGS and MKN45 cells, flow cytometry analysis was performed in this study. Initially, MKN45 and AGS cells were cultivated for 24 h after being planted at a density of 1 × 10^4^ cells per well in 24-well plates. Following dissociation with 0.25% trypsin-EDTA and washing with 1 × Phosphate-buffered saline (PBS, pH 7.4), cells were cultured with 5 μL Annexin V-FITC solution and 5 μL of propidium iodide (PI) for 15 min at room temperature for apoptosis analysis. Subsequently, a BD FACSCalibur flow cytometer (Becton, Dickinson and Company, Franklin Lakes, NJ, USA) was used to assess the staining of PI and Annexin V, and FlowJo software (version 10.6.0, FlowJo software, Ashland, OR, USA) was used to analyze the results. For cell cycle analysis, MKN45 and AGS cells were fixed for the whole night at 4°C in 75% ice-cold ethanol. After washing with PBS, cells were treated for 30 min at 37°C in the dark with 50 μg/mL PI containing 10 mg/L RNase A. A BD FACSCalibur flow cytometer (Becton, Dickinson and Company, Franklin Lakes, NJ, USA) was utilized to assess the cell cycle distribution, and FlowJo software (version 10.6.0, FlowJo software, Ashland, OR, USA) was applied to analyze the information.

### 2.11 Cell Counting Kit-8 (CCK-8) Assay

The Cell Counting Kit-8 (CCK-8) assay (CK04, Dojindo, Kumamoto, Japan) was applied to evaluate cell proliferation. For time-course experiments, cells (MKN45 and AGS) were seeded at a density of 1 × 10^3^ cells/well in 96-well plates and cultured for 1, 2, 3, and 4 days. At each time point, 10 μL of CCK-8 solution was added to each well, and cells were incubated for 1 h at 37°C. For endpoint viability assays, cells were cultured for 24 h after different treatments before adding CCK-8 solution. Absorbance at 450 nm was measured using a microplate reader (Multiskan GO, Thermo Fisher Scientific, Waltham, MA, USA).

### 2.12 Colony Formation Assay

Colony formation was measured using the plate colony formation assay. Briefly, 200 cells (MKN45 and AGS) from each group were cultivated for 10 days in DMEM with 10% FBS after being planted into each well of a 6-well plate. After incubation, cells were washed with 1× PBS (pH 7.4) and fixed with 0.1% alkaline nitroblue tetrazolium chloride. Colony images were captured, and colonies were counted under a microscope (Model AE31, Motic, Xiamen, China).

### 2.13 RNA Sequencing and Differential Expression Analysis of MKN45 Cells Transfected with si-CENPF-1

After transfecting MKN45 cells with si-NC or si-*CENPF*-1, cells were incubated for 24 h, followed by RNA extraction. To ensure reproducibility, three biological replicates of each treatment group were conducted. Following the manufacturer’s instructions, TRIzol reagent (Tiangen, Beijing, China) has been utilized to isolate RNA. An Agilent 2100 Bioanalyzer (Agilent, Shanghai, China) was applied to evaluate the RNA’s integrity, with RNA integrity number (RIN) > 8.0 confirmed for all samples; a NanoDrop 2000 spectrophotometer (Thermo Scientific, Waltham, MA, USA) was performed to determine the RNA’s concentration and purity. The VAHTS Universal V6 RNA-seq Library Prep Kit (Vazyme, Nanjing, China) produced RNA sequencing libraries according to the manufacturer’s guidelines. Sequencing was implemented on the Illumina NovaSeq 6000 platform (Illumina, Inc., San Diego, California, USA), providing high-throughput and high-quality data with a genome mapping rate > 95% for all samples. Shanghai Meiji Biopharmaceutical Technology Co., Ltd., conducted all library preparation and sequencing procedures.

### 2.14 Related Bioinformatics Analysis of Sequencing Data

Genes in the sequencing data were screened according to the FC threshold (greater than 2 for up-regulation and less than 0.5 for down-regulation), and the statistical significance standard was *p* < 0.05. DEGs identified from sequencing data were subjected to Gene Ontology (GO) and Kyoto encyclopedia of genes and genomes (KEGG) pathway analysis utilizing the Database for Annotation, Visualization, and Integrated Discovery (DAVID) platform (https://davidbioinformatics.nih.gov/summary.jsp). Gene Set Enrichment Analysis (GSEA) was conducted for the candidate gene *CENPF* by the Sangerbox platform (http://vip.sangerbox.com/home.html) to identify enriched signaling pathways. Subsequently, to explore the interaction relationship between CENPF, MYC, and GLS, we constructed a gene interaction network using the Search Tool for the Retrieval of Interacting Genes database (STRING: https://string-db.org/). In addition, the correlation between *CENPF* and c-*Myc* was also detected. Correlation analysis was performed using the “ggstatsplot” package (version 0.9.5) (https://CRAN.R-project.org/package=ggstatsplot) to evaluate the correlation between non-normally distributed quantitative variables.

### 2.15 Chromatin Immunoprecipitation (ChIP)

ChIP studies were conducted on MKN45 and AGS cell line samples by the Millipore EZ ChIP^™^ Chromatin Immunoprecipitation Kit (26156, Thermo Fisher Scientific, Waltham, MA, USA), in accordance with the manufacturer’s instructions. Cross-linked chromatin DNA was, in short, sonicated into segments of 200–500 bp. Primary antibodies (anti-c-Myc, ab32072, Abcam, Cambridge, UK) were used at a dilution of 1:100, and normal IgG were used to immunoprecipitate the fragments. Protein G agarose beads were used to collect, clean, and rinse the DNA-protein complexes, and the cross-links were reversed. The enrichment of c-Myc at the GLS site (containing the CACGTG motif) was quantified by RT-qPCR utilizing SYBR Green PCR Master Mix (Takara, Kameoka, Japan). Results were calculated as fold enrichment relative to IgG control.

### 2.16 Luciferase Reporter Assay

The predicted *GLS* promoter region was amplified and cloned into the pGL3-Basic luciferase reporter vector (Promega, Madison, WI, USA). The wild-type (*GLS* WT) or mutated (*GLS* MUT) luciferase reporter vectors, along with siRNA for CENPF-1 (si-*CENPF*-1), empty vector, or overexpression plasmid for *c-Myc* (over-*c-Myc*), were co-transfected into MKN45 and AGS cells by Lipofectamine 2000 (Invitrogen, Carlsbad, CA, USA). The Luciferase Assay Kit (Promega, Madison, WI, USA) was employed to lyse the cells after 48 h, and a Luminometer (Berthold Lumat LB 9507, Berthold Technologies, Germany) was applied to assess the luciferase activity. The results were normalized to control groups and presented as relative luciferase activity.

### 2.17 Measuring Glutamine Consumption, Glutamate Production, and **α**-Ketoglutarate (**α**-KG) Levels in GC Cells

The Glutamine Assay Kit, Glutamate Assay Kit, and α-Ketoglutarate Assay Kit were applied in accordance with the manufacturer’s instructions to assess glutamine intake, glutamate production, and α-ketoglutarate levels. The Glutamine Assay Kit (colorimetric method) and Glutamate Assay Kit (colorimetric method) were obtained from Beijing Baiaolaibo Technology Co., Ltd., while the α-Ketoglutarate Assay Kit was obtained from Abnova (Shanghai, China). Briefly, MKN45 and AGS cells were cultured 2 × 10^5^ cells per well in 6-well plates, and the culture medium was collected for analysis. The concentrations of glutamine, glutamate, and α-ketoglutarate were determined by employing a Microplate Reader (Thermo Fisher Scientific, Shanghai, China) to measure absorbance at 570 nm.

To ensure the robustness of our metabolic assays to batch effects, several measures were taken. The assays were conducted in a controlled environment with standardized protocols to minimize variability. Additionally, internal standards and controls were included in each batch to monitor and adjust for any potential batch effects. The data from the metabolic assays were analyzed using appropriate statistical methods, and the results were validated through multiple independent experiments.

### 2.18 Relative Antioxidant Capacity Detection of GC Cells

The ratio of glutathione (GSH)/glutathione disulfide (GSSG) in GC cells was measured using the GSH/GSSG Assay Kit (Beyotime, Shanghai, China) according to the manufacturer’s instructions. The cells (MKN45 and AGS) were collected and lysed in the protein removal buffer M provided in the kit. Cell lysis was achieved by three freeze-thaw cycles with liquid nitrogen and 37°C water. The lysate was centrifuged at 12,000 rpm for 15 min at 4°C, and the collected supernatant was used for GSH and GSSG detection.

### 2.19 Statistical Analysis

All experimental data are presented as the mean ± standard deviation (SD) from at least three independent biological replicates. Statistical analysis was performed utilizing the R programming language (version 4.3.1). For comparisons between two groups, an unpaired two-tailed Student’s *t*-test was used. For comparisons among more than two groups, one-way analysis of variance (ANOVA) followed by Tukey’s multiple comparison test was applied. When two or more factors were involved, two-way ANOVA with appropriate post-hoc testing was used to evaluate interaction effects. The Shapiro–Wilk test was performed to verify data normality, and Levene’s test was applied to assess homogeneity of variance. Nonparametric tests (e.g., Mann–Whitney U test or Kruskal–Wallis test) were used when assumptions of normality or equal variance were not met. Statistical significance was defined as a *p* < 0.05.

## 3 Results

### 3.1 Differential Expression and Protein-Protein Interaction (PPI) Network Analyses Reveal Key Candidate Genes in GC

**Figure 1:**
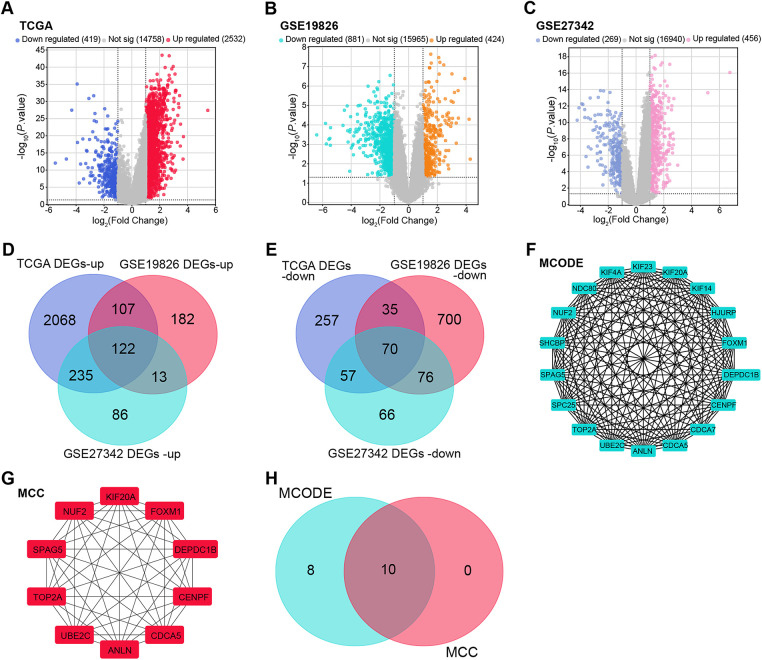
DEG analysis and PPI network construction across multiple datasets. (**A**) Volcano plot depicting the DEGs in the TCGA-STAD dataset, with upregulated genes shown in red and downregulated genes in dark blue. (**B**) Volcano plot illustrating the DEGs in the GSE19826 dataset, with upregulated genes in orange and downregulated genes in green. (**C**) Volcano plot representing the DEGs in the GSE27342 dataset, with upregulated genes in pink and downregulated genes in light blue. (**D**) Venn diagram showing the overlapping upregulated DEGs across the TCGA-STAD, GSE19826, and GSE27342 datasets. (**E**) Venn diagram displaying the overlapping downregulated DEGs across the TCGA-STAD, GSE19826, and GSE27342 datasets. (**F**) PPI network constructed for the overlapping DEGs using the MECODE algorithm. The network includes 18 nodes and 149 edges, where each node represents a protein and each edge represents a predicted protein-protein interaction. (**G**) Identification of the top 10 interacting genes from the PPI network of overlapping DEGs based on the MCC algorithm, with 10 nodes and 45 edges. (**H**) Venn diagram showing the overlapping genes between the MECODE and MCC algorithm networks. DEG: differentially expressed gene; TCGA-STAD: the cancer genome atlas—stomach adenocarcinoma; PPI: protein-protein interaction; MCODE: molecular complex detection; MCC: maximal clique centrality

To clarify the molecular mechanisms underlying GC, differential expression analysis was performed across multiple datasets. In the TCGA-STAD dataset, 2532 upregulated and 419 downregulated DEGs were detected (Fig. 1A). Analysis of the GSE19826 dataset revealed 424 upregulated and 881 downregulated DEGs, while the GSE27342 dataset identified 456 upregulated and 269 downregulated DEGs (Fig. 1B,C). Topological analysis of DEGs across these datasets revealed 122 overlapping upregulated DEGs and 70 overlapping downregulated DEGs (Fig. 1D,E). PPI networks were constructed using the MECODE and MCC algorithms to investigate interactions among the overlapping DEGs further. The MECODE algorithm yielded a network comprising 18 nodes and 149 edges, while the MCC algorithm identified a network with 10 nodes and 45 edges (Fig. 1F,G). Integration of these PPI networks and subsequent topological analysis led to the identification of 10 highly associated candidate genes (Fig. 1H).

### 3.2 Expression Analysis and Predictive Performance Evaluation of Candidate Genes Identify CENPF as the Hub Gene

**Figure 2:**
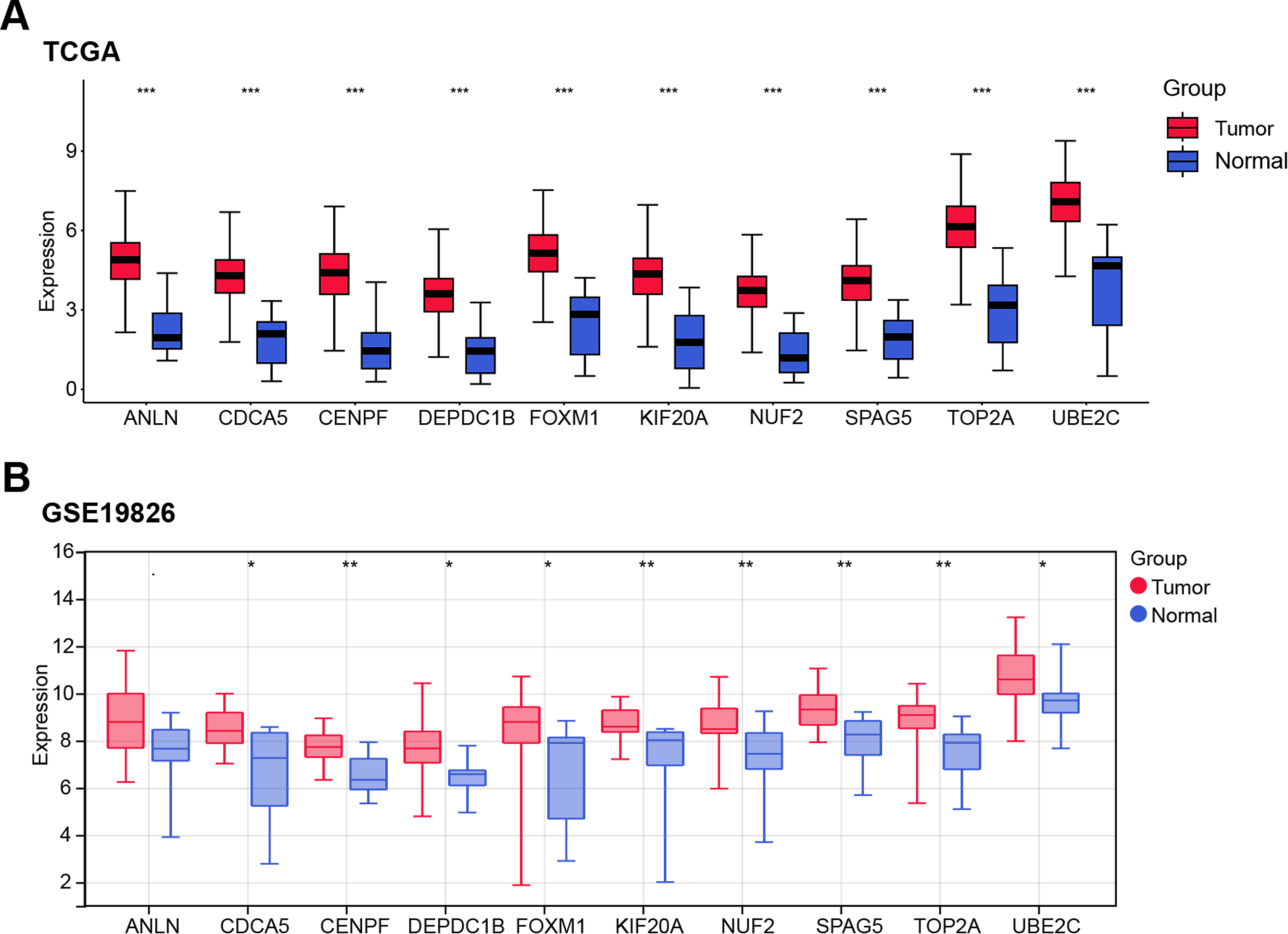

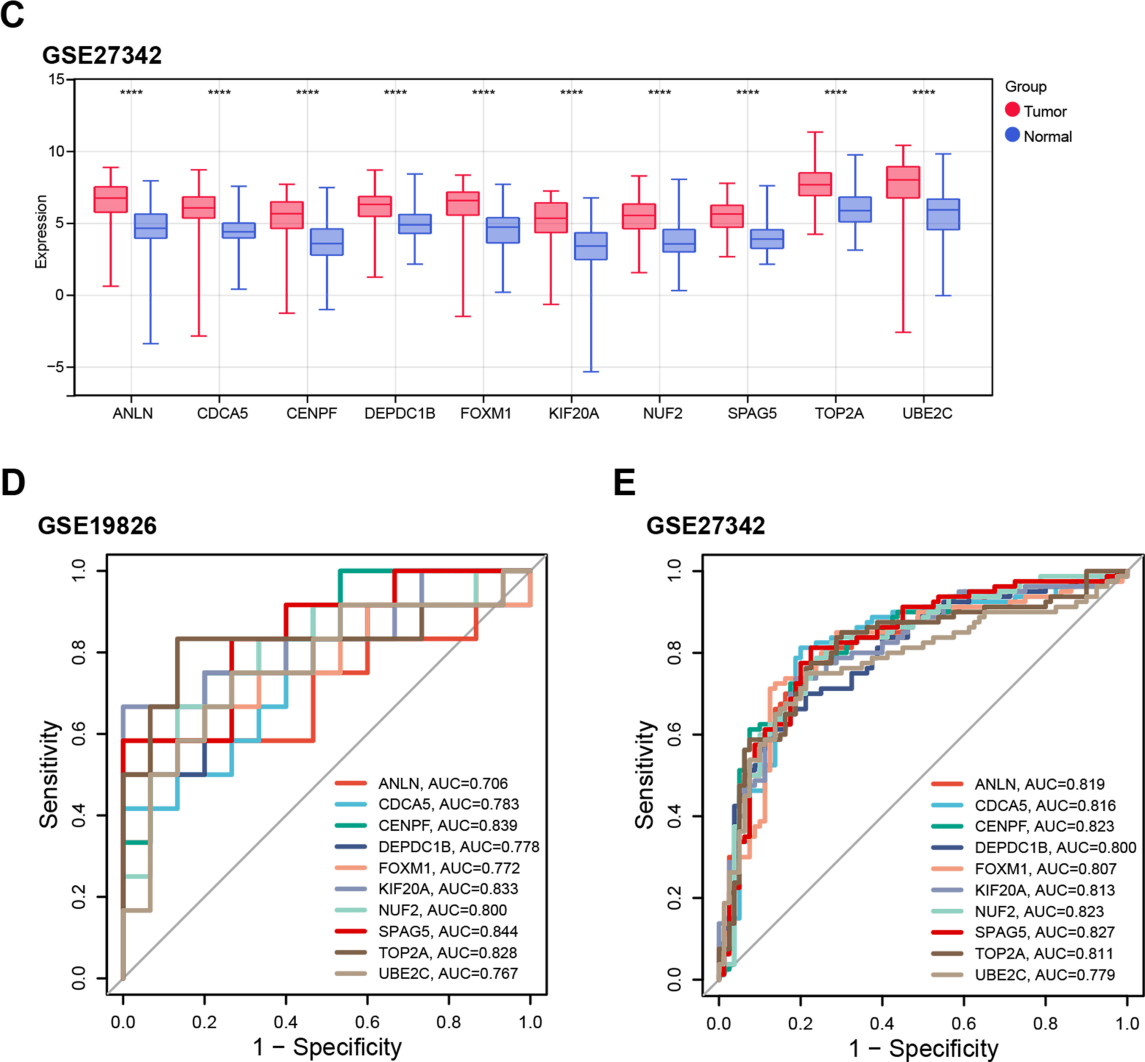
Expression patterns and predictive performance of candidate genes in GC. (**A**) Boxplot showing the expression levels of ten candidate genes (*ANLN*, *CDCA5*, *CENPF*, *DEPDC1B*, *FOXM1*, *KIF20A*, *NUF2*, *SPAG5*, *TOP2A*, *UBE2C*) in tumor and normal tissues from the TCGA-STAD dataset. (**B**) Boxplot displaying the expression levels of the ten candidate genes in tumor and normal tissues from the GSE19826 dataset. (**C**) Boxplot illustrating the expression levels of the ten candidate genes in tumor and normal tissues from the GSE27342 dataset. (**D**) ROC curve assessing the diagnostic accuracy of the ten candidate genes in the GSE19826 dataset. The AUC value for each gene is provided. (**E**) ROC curve evaluating the diagnostic accuracy of the ten candidate genes in the GSE27342 dataset, with AUC values listed for each gene. GC: gastric cancer; TCGA-STAD: the cancer genome atlas—stomach adenocarcinoma; ROC: receiver operating characteristic; AUC: area under the curve. *p < 0.05 or **p < 0.01 or ***p < 0.001 or ****p < 0.0001 vs. Tumor

Expression levels of the ten candidate genes (*ANLN*, *CDCA5*, *CENPF*, *DEPDC1B*, *FOXM1*, *KIF20A*, *NUF2*, *SPAG5*, *TOP2A*, *UBE2C*) were analyzed across tumor and normal tissues in the TCGA-STAD, GSE19826, and GSE27342 datasets. All ten genes were markedly elevated in tumor tissues vs. normal tissue (Fig. 2A–C). In the GSE19826 dataset, *CENPF* (AUC = 0.839) and *SPAG5* (AUC = 0.844) demonstrated high predictive accuracy (Fig. 2D), while in the GSE27342 dataset, *CENPF* (AUC = 0.823) and *SPAG5* (AUC = 0.827) also exhibited strong performance (Fig. 2E). While *FOXM1*, *SPAG5*, and *NUF2* all showed upregulation in GC tissues, *CENPF* exhibited more stable diagnostic performance across these independent datasets: its AUC values were comparable to *SPAG5* in both GSE19826 and GSE27342, but outperformed *FOXM1* (GSE19826: 0.772; GSE27342: 0.807) and NUF2 (GSE19826: 0.800; GSE27342: 0.823) in cross-dataset reproducibility—supporting its potential as a more robust GC-related marker. Since the relevant mechanism of *SPAG5* in cancer metabolic reprogramming is still unclear, and *CENPF* has been shown to be a gene related to cancer metabolic reprogramming in some existing studies [20], this study selected *CENPF* as a hub gene for further research on GC.

### 3.3 Upregulation of CENPF in GC Cells Promoted Cell Proliferation and Viability

**Figure 3:**
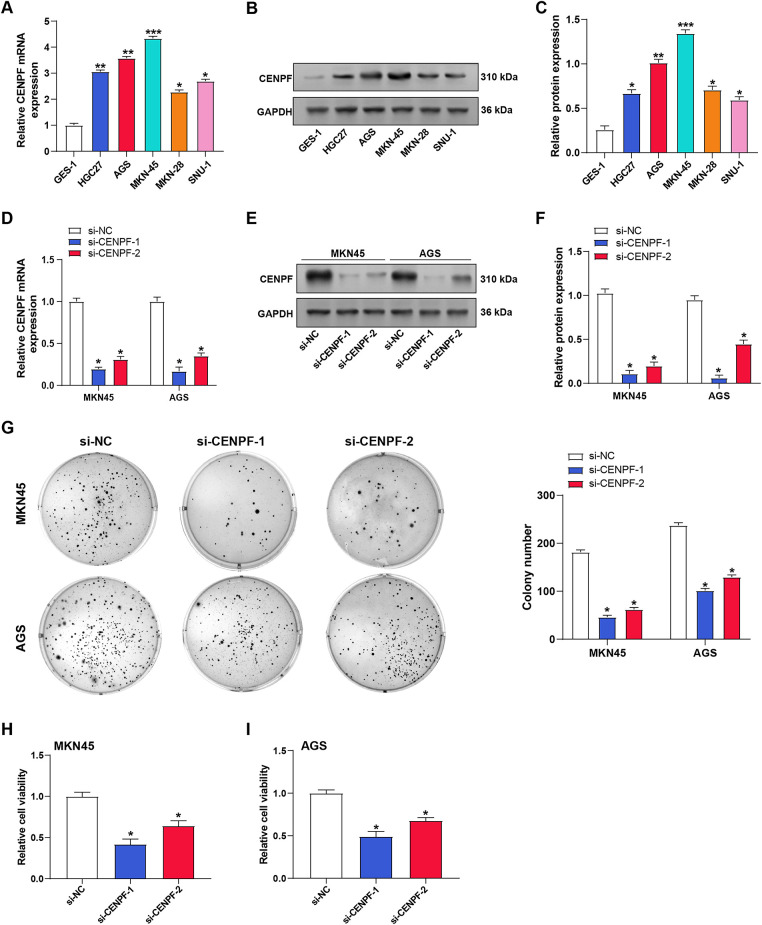
Upregulation of *CENPF* in GC cells and its role in proliferation and viability. (**A**) Relative mRNA expression levels of *CENPF* in normal gastric epithelial cells (GES-1) and GC cell lines (HGC27, AGS, MKN45, MKN28, SNU1) as determined by RT-qPCR. (**B**,**C**) Protein expression levels of CENPF in normal gastric epithelial cells (GES-1) and GC cell lines (HGC27, AGS, MKN45, MKN28, SNU1) as determined by WB, with quantification shown in the bar graphs. (**D**) RT-qPCR analysis confirming the knockdown efficiency of *CENPF* using siRNA (si-*CENPF*-1, si-*CENPF*-2) or negative control siRNA (si-NC) in MKN45 and AGS cells. (**E**,**F**) WB validation of CENPF knockdown efficiency in MKN45 and AGS cells transfected with si-*CENPF*-1 or si-*CENPF*-2, with quantification shown in the bar graphs. (**G**) Representative colony formation assay demonstrating the effect of *CENPF* knockdown on MKN45 and AGS cell proliferation, with colony numbers quantified. (**H**,**I**) CCK-8 assessed cell viability after transfection with si-NC, si-*CENPF*-1, or si-*CENPF*-2 in MKN45 (**H**) and AGS (**I**) cells. OD values at 450 nm are shown. GC: gastric cancer; RT-qPCR: quantitative reverse transcription polymerase chain reaction; siRNA: small interfering RNA; CCK-8: cell counting kit-8; OD: optical density. *p < 0.05 or **p < 0.01 or ***p < 0.001 vs. GES-1. *p < 0.05 vs. si-NC

The expression of *CENPF* was examined in GC cell lines (HGC27, AGS, MKN45, MKN28, and SNU1) and normal gastric epithelial cells (GES-1) by RT-qPCR (Fig. 3A) and WB (Fig. 3B,C). The findings showed that *CENPF* expression was substantially higher in GC cell lines compared to normal cells. Among the cell lines tested, MKN45 and AGS showed the most pronounced upregulation of CENPF, and were selected for subsequent experiments. To assess the effectiveness of two siRNAs that target *CENPF* (si-*CENPF*-1 and si-*CENPF*-2), RT-qPCR and WB were performed after transfection into MKN45 and AGS cells. Both assays confirmed the successful knockdown of *CENPF* expression in these cells (Fig. 3D–F). Functional assays were executed to assess the impact of *CENPF* knockdown on cell phenotypes. Colony formation assays revealed that silencing *CENPF* inhibited the proliferative capacity of AGS and MKN45 cells (Fig. 3G). Moreover, CCK-8 assays revealed a substantial reduction in cell viability in both cell lines following *CENPF* knockdown (Fig. 3H,I). These findings strongly demonstrated that *CENPF* is upregulated in GC cells and stimulates cell proliferation.

### 3.4 CENPF Knockdown Promotes Apoptosis and Induces G2 Phase Arrest in GC Cells

**Figure 4:**
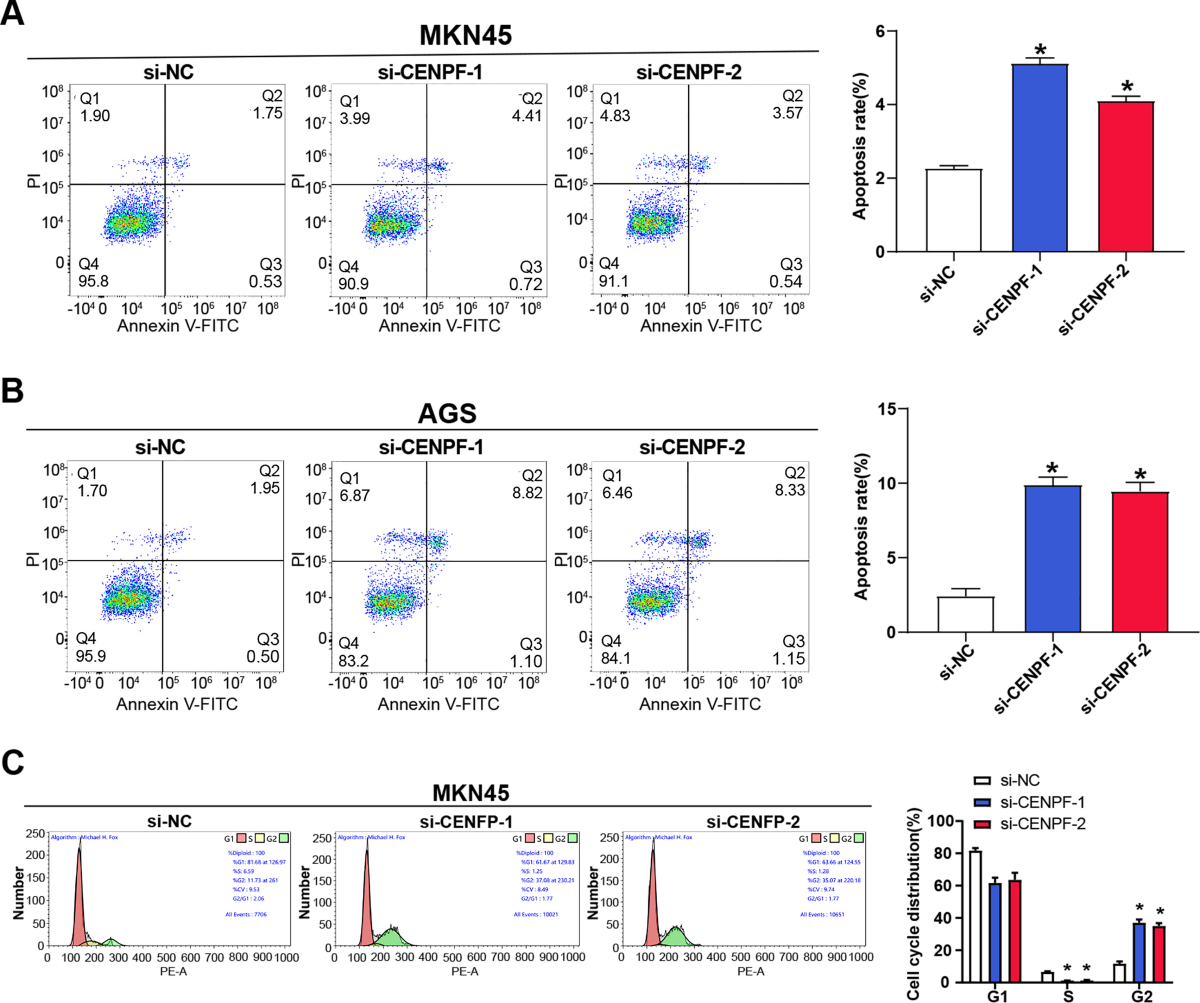

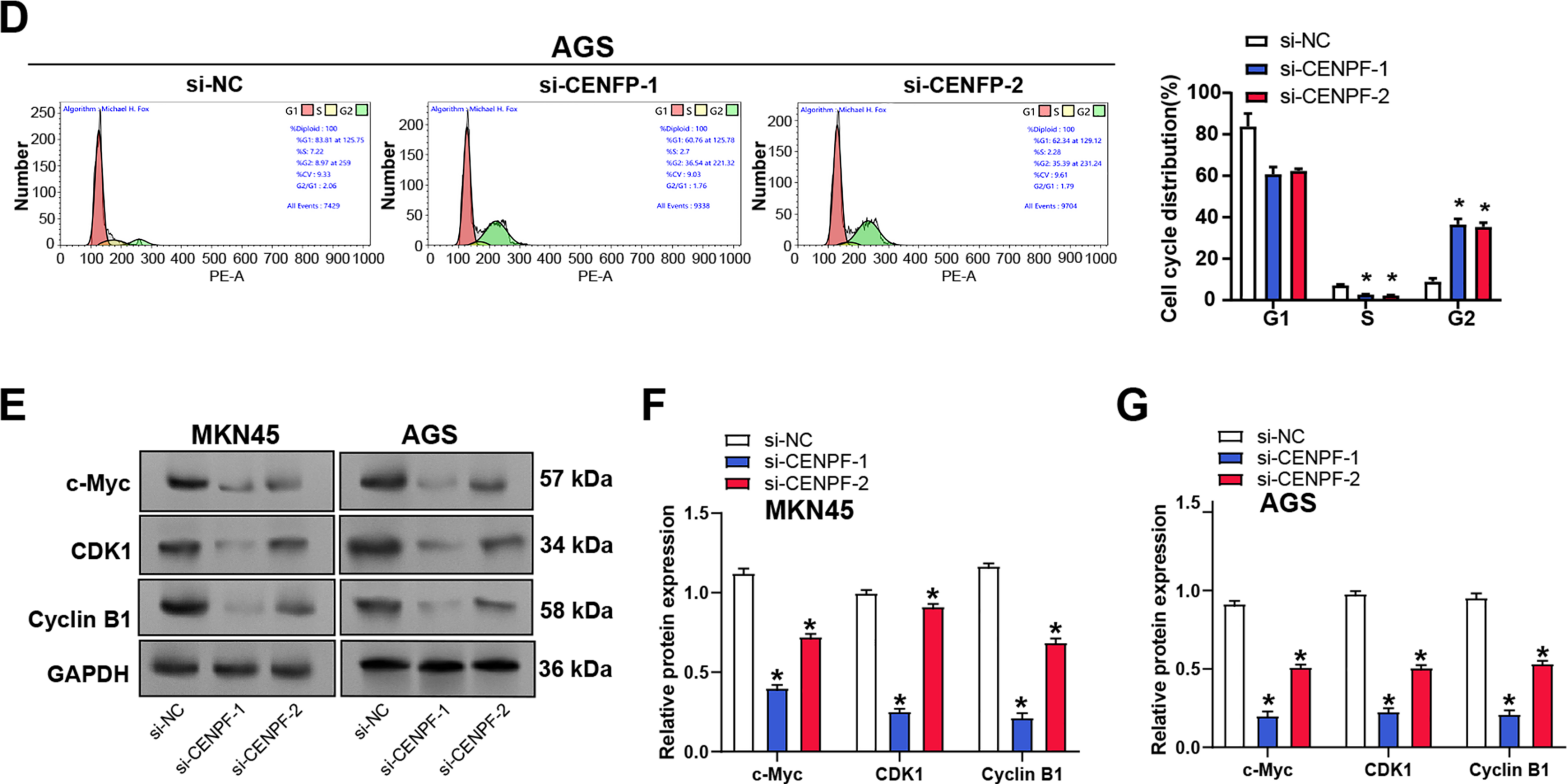
Effects of *CENPF* knockdown on apoptosis, cell cycle progression, and tumor growth in GC. (**A**,**B**) Flow cytometry analysis of apoptosis in MKN45 (**A**) and AGS (**B**) cells after *CENPF* knockdown. (**C**,**D**) Flow cytometry analysis of cell cycle distribution in MKN45 (**C**) and AGS (**D**) cells after *CENPF* knockdown. (**E**–**G**) WB analysis of c-Myc and CDK1 protein levels in MKN45 (**E**) and AGS (**F**) cells after *CENPF* knockdown, with quantification shown in the bar graphs. GC: gastric cancer. *p < 0.05 vs. si-NC.

To further explore the function of *CENPF* in GC cell behavior, flow cytometry was performed to analyze its impact on apoptosis and cell cycle in AGS and MKN45 cells. The outcomes showed that *CENPF* knockdown significantly promoted apoptosis in AGS and MKN45 cell lines in contrast to the control group, although the changes were modest (Fig. 4A,B). Additionally, flow cytometry revealed a decreased proportion of cells in the G2 phase, indicating a cell cycle arrest at the G2 phase (Fig. 4C,D). WB was conducted to examine key cell cycle-associated protein expression in MKN45 and AGS cells following *CENPF* knockdown. The outcomes illustrated a decrease in the levels of c-Myc and CDK1 in both cell lines (Fig. 4E–G), suggesting that *CENPF* reduced GC cell apoptosis and encouraged cell cycle progression.

### 3.5 CENPF Influences Amino Acid Metabolism and Correlates with the Citrate Cycle and Glutamine Pathway

**Figure 5:**
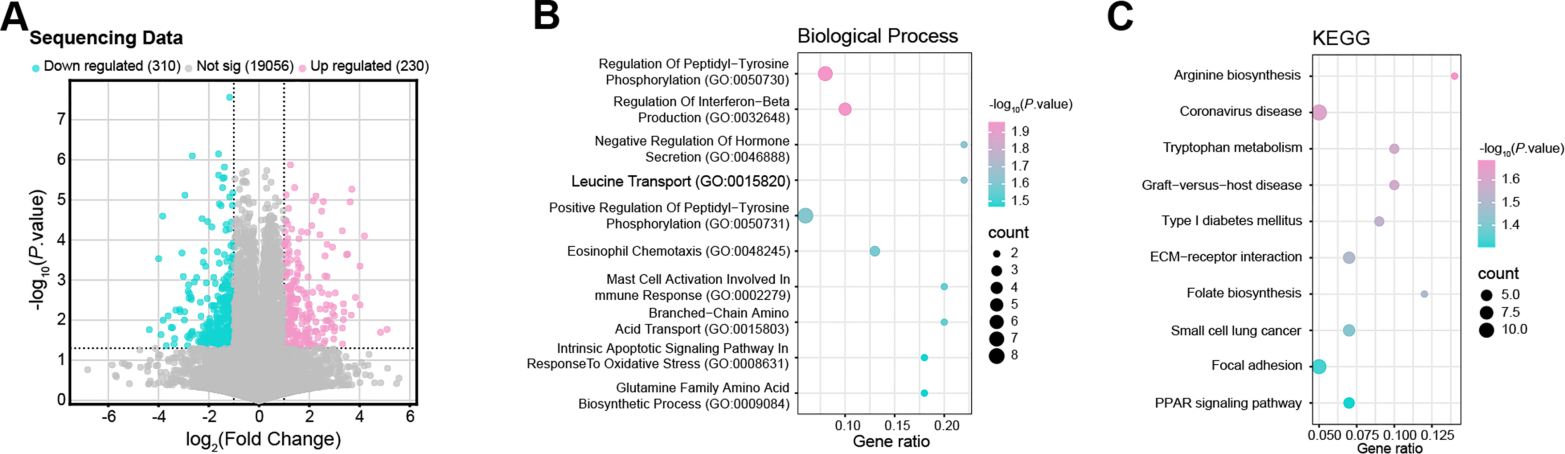

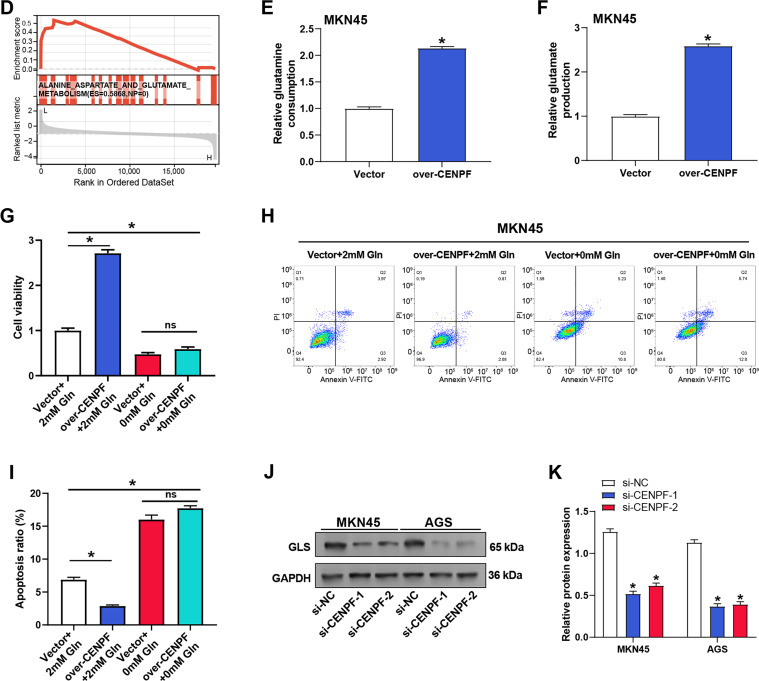
*CENPF* influences amino acid metabolism and correlates with key metabolic pathways. (**A**) Volcano plot showing the DEGs between MKN45 cells transfected with si-*CENPF* and si-NC. Upregulated genes (pink) and downregulated genes (green) were identified based on *p*-value and log_2_ fold change thresholds. (**B**) GO enrichment analysis of the function of DEGs based on BP. The abscissa is GeneRatio, and the ordinate is an enrichment term. The larger the dots, the more genes are enriched. (**C**) KEGG enrichment analysis predicted the pathways of DEGs involvement. (**D**) GSEA indicates a significant association between *CENPF* and the signal pathway. The ES indicates the strength of the pathway association with *CENPF* expression. (**E**) Glutamine consumption assay demonstrating the effect of *CENPF* overexpression on glutamine consumption in MKN45 cells. *p < 0.05 vs. Vector group. (**F**) Glutamate production assay showing the impact of *CENPF* overexpression on glutamate production in MKN45 cells. *p < 0.05 vs. Vector group. (**G**) CCK-8 assay assessing cell viability under different conditions: normal culture (2 mM Gln), *CENPF* overexpression with 2 mM Gln, and *CENPF* overexpression with glutamine depletion (0 mM Gln). ns: not significant. *p < 0.05 vs. Vector + 2 mM Gln. (**H**,**I**) Flow cytometry analysis of apoptosis in MKN45 cells under normal culture conditions (2 mM Gln), *CENPF* overexpression with 2 mM Gln, and *CENPF* overexpression with glutamine depletion (0 mM Gln). ns: not significant. *p < 0.05 vs. Vector + 2 mM Gln. (**J**,**K**) WB analysis of GLS expression in MKN45 and AGS cells following *CENPF* knockdown under normal culture conditions, with quantitative data represented in bar graphs. DEG: differentially expressed gene; GO: gene ontology; BP: biological process; KEGG: Kyoto encyclopedia of genes and genomes; GSEA: gene set enrichment analysis; ES: enrichment score; CCK-8: cell counting kit-8; Gln: glutamine; GLS: glutaminase. *p < 0.05 vs. si-NC

RNA sequencing of MKN45 cells transfected with either si-NC or si-*CENPF* identified 230 upregulated DEGs and 310 downregulated DEGs (Fig. 5A). BP analysis highlighted that the DEGs were enriched in terms such as “Branched-Chain Amino Acid Transport (GO:0015803)”, “Leucine Transport (GO:0015820)”, and “Glutamine Family Amino Acid Biosynthetic Process (GO:0009084)” (Fig. 5B). KEGG pathway analysis identified significant enrichment of DEGs in pathways such as “Arginine Biosynthesis”, “Tryptophan Metabolism”, and “Folate Biosynthesis” (Fig. 5C). Moreover, GSEA revealed a strong association between *CENPF* and the “Alanine, Aspartate, and Glutamate Metabolism” pathway (Fig. 5D). Overall, these bioinformatics analyses indicate that *CENPF* may be crucial for regulating amino acid metabolism in GC cells, with particular emphasis on the glutamine metabolic pathway.

### 3.6 CENPF Regulates GC Proliferation and Apoptosis through Glutamine Metabolism

To better understand the function of *CENPF* in glutamine metabolism in GC, measurements of glutamine intake and glutamate synthesis were performed by specific detection kits. The results showed that *CENPF* knockdown reduced glutamine consumption and glutamate production in AGS cells compared with the si-NC group (Fig. A1B,C). Conversely, to evaluate whether *CENPF* overexpression could enhance glutamine metabolism, we established *CENPF* overexpression cell lines in MKN45 and AGS cells. Successful overexpression of *CENPF* was verified at the protein and mRNA levels (Fig. A1D–F). To further investigate the functional significance of this metabolic regulation. Specific detection kits were employed to assess glutamate synthesis and glutamine intake. *CENPF* overexpression increased glutamine consumption and glutamate production in MKN45 cells compared with the vector group (Fig. 5E,F). Further analysis using CCK-8 assays disclosed that *CENPF* overexpression improved cell viability of MKN45 cells under normal culture conditions (2 mM Gln). However, under glutamine-deficient conditions (0 mM Gln), the viability of MKN45 cells overexpressing *CENPF* was lower than that of cells grown on normal media (Fig. 5G). Flow cytometry analysis showed that overexpression of *CENPF* reduced apoptosis of MKN45 cells cultured in normal medium (2 mM Gln), while glutamine deficiency reversed this effect and induced apoptosis (Fig. 5H,I). Subsequently, the ratio of intracellular GSH and GSSG under different conditions showed that in normal culture medium (2 mM Gln), overexpression of *CENPF* promoted the increase of the GSH/GSSG ratio and improved cellular antioxidant capacity. However, after glutamine deprivation, the ratio of Vector + 0 mM Gln to Vector + 2 mM Gln decreased, and there was no significant change after overexpression of *CENPF* compared with Vector + 0 mM Gln (Fig. A1G). WB analysis confirmed that *CENPF* knockdown inhibited the levels of GLS in MKN45 and AGS cells cultured in normal medium compared with the si-NC group (Fig. 5J,K). These outcomes suggest that *CENPF* may modulate GC cell proliferation and apoptosis by regulating glutamine metabolism.

### 3.7 CENPF Regulates GC Cell Proliferation through GLS-Mediated Glutamine Metabolism

**Figure 6:**
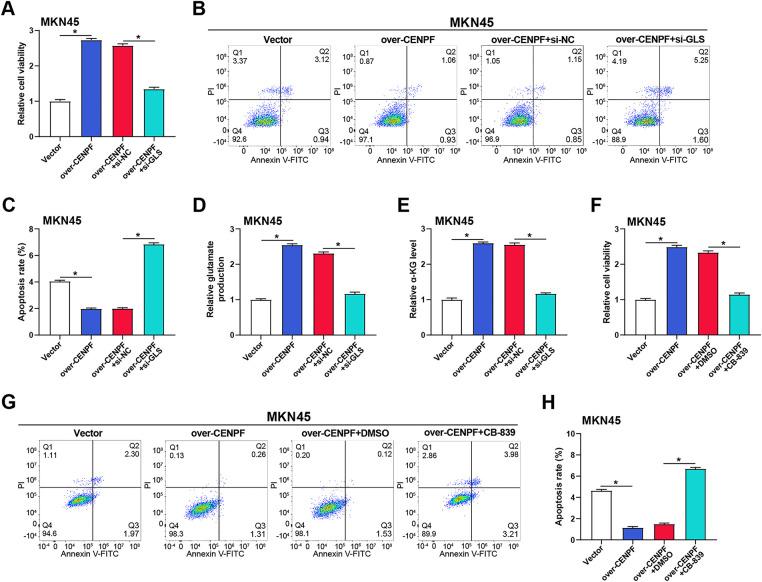
*CENPF* promotes glutamine metabolism and regulates cell proliferation through *GLS*. (**A**) CCK-8 assay showing the impact of *CENPF* overexpression and/or *GLS* knockdown on MKN45 cell viability. (**B**,**C**) Flow cytometry analysis of apoptosis in MKN45 cells with *CENPF* overexpression and/or *GLS* knockdown, with quantitative data shown in the bar graph. (**D**) Glutamate production assay assessing the effect of *CENPF* overexpression and/or *GLS* knockdown on glutamate levels in MKN45 cells. (**E**) α-KG assay showing the impact of *CENPF* overexpression and/or *GLS* knockdown on α-KG levels in MKN45 cells. (**F**) CCK-8 assay evaluating the effect of *CENPF* overexpression and/or selective GLS inhibitor Telaglenastat (CB-839) on MKN45 cell viability. (**G**,**H**) Flow cytometry analysis of apoptosis in MKN45 cells with *CENPF* overexpression and/or CB-839 treatment, with quantitative data presented in the bar graph (**H**). *p < 0.05 vs. Vector or over-*CENPF* + si-NC

**Figure A2:**
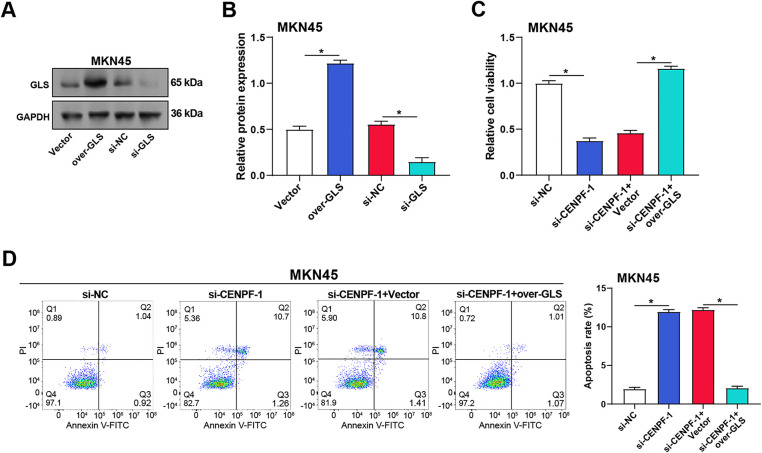
**Effect of *CENPF* knockdown and *GLS* overexpression on cell viability, apoptosis, and *GLS* expression** **in MKN45 cells.** (**A**,**B**) WB analysis confirming the efficiency of *GLS* overexpression and knockdown in MKN45 cells, with quantitative results shown in the bar graphs. (**C**) CCK-8 assay evaluating the impact of *CENPF* knockdown and *GLS* overexpression on MKN45 cell viability. (**D**) Flow cytometry analysis assessing the effect of *CENPF* knockdown and *GLS* overexpression on apoptosis in MKN45 cells, with quantitative data presented in the bar graph. CCK-8: cell counting kit-8. *p < 0.05 vs. Vector or si-NC or si-*CENPF*-1 + Vector

The interaction between *CENPF* and glutamine metabolism in GC cells was further investigated by evaluating the effects of *GLS*, glutamate, and α-KG in MKN45 cells. WB analysis was applied to confirm the overexpression and knockdown efficiency of GLS in MKN45 cells (Fig. A2A,B). Functional experiments showed that *CENPF* knockdown caused apoptosis and suppressed cell proliferation, while *GLS* overexpression treatment reversed this effect (Fig. A2C,D). In contrast, CCK-8 experiments revealed that *CENPF* overexpression promoted cell proliferation, while co-treatment of overexpressed *CENPF* combined with si-*GLS* reduced this effect (Fig. 6A). Flow cytometry analysis showed that overexpression of *CENPF* inhibited apoptosis in MKN45 cells, while si-*GLS* reversed this protective effect against apoptosis (Fig. 6B,C). Subsequent analysis using relevant detection kits demonstrated that *CENPF* overexpression increased the production of glutamate and α-KG, while co-treatment with si-*GLS* inhibited these effects (Fig. 6D,E). To further confirm the function of *GLS* in *CENPF*-mediated effects, we applied CB-839 (a specific GLS inhibitor) to cells that overexpressed *CENPF*. Consistent with the knockdown results, pharmacological inhibition of *GLS* by CB-839 decreased cell viability and promoted apoptosis in *CENPF*-overexpressing cells (Fig. 6F–H).

### 3.8 CENPF Stabilizes c-Myc by Modulating the Ubiquitin-Proteasome System in GC Cells

**Figure 7:**
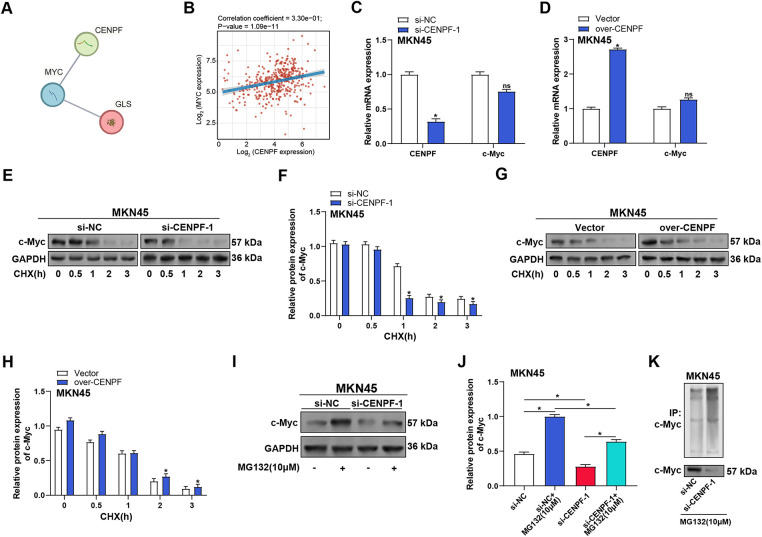
CENPF stabilizes c-Myc by modulating the ubiquitin-proteasome system in GC cells. (**A**) PPI network analysis showing the interactions between *CENPF*, *c-Myc*, and *GLS*. (**B**) Correlation analysis based on the TCGA-STAD dataset showing the correlation between *CENPF* and *c-Myc*. (**C**,**D**) RT-qPCR analysis of the effects of *CENPF* knockdown (**C**) or overexpression (**D**) on the mRNA expression levels of *CENPF* and *c-Myc* in MKN45 cells. (**E**,**F**) WB analysis of c-Myc degradation in MKN45 cells following *CENPF* knockdown and CHX treatment for the indicated times (0, 0.5, 1, 2, and 3 h). (**G**,**H**) WB analysis of c-Myc degradation in MKN45 cells overexpressing *CENPF* after CHX treatment at various time points. (**I**,**J**) WB analysis of the effects of proteasome inhibitor MG132 and *CENPF* knockdown on c-Myc protein expression levels in MKN45 cells, with quantification shown in panel (**J**). (**K**) Immunoprecipitation analysis of c-Myc ubiquitination in MKN45 cells with *CENPF* knockdown under MG132 treatment. GC: gastric cancer; PPI: protein-protein interaction; TCGA-STAD: the cancer genome atlas-stomach adenocarcinoma; RT-qPCR: quantitative reverse transcription polymerase chain reaction; CHX: cycloheximide. ns: not significant. *p < 0.05 vs. si-NC or si-NC + MG132 or si-*CENPF*-1

According to reports, the protein known as the oncogenic transcription factor c-Myc has a short lifespan, typically degraded through the ubiquitin-proteasome pathway. c-Myc is located upstream of the glutaminolysis pathway and has been shown to directly regulate key enzymes in the glutaminolysis pathway, including GLS, which catalyzes the conversion of glutamine to glutamate [21]. Therefore, we explored whether *CENPF* regulates *c-Myc* in GC cells via proteasome-dependent degradation. A further study employing a PPI network analysis revealed interactions among *CENPF*, *c-Myc*, and *GLS* (Fig. 7A). Correlation analysis based on the TCGA-STAD dataset showed a positive correlation between *CENPF* and *c-Myc* (*p* = 1.09 × 10^−11^, r = 0.33), suggesting a potential cooperative role in GC progression (Fig. 7B). RT-qPCR analysis of MKN45 cells revealed that *CENPF* knockdown or overexpression did not significantly affect *c-Myc* mRNA levels, indicating a non-transcriptional regulation mechanism (Fig. 7C,D). Using CHX to assess c-Myc degradation over time (0, 0.5, 1, 2, 3 h), WB analysis demonstrated that *CENPF* knockdown accelerated c-Myc degradation, while *CENPF* overexpression prolonged its half-life (Fig. 7E–H). To investigate the role of the proteasome pathway, *CENPF* knockdown MKN45 cells were treated with MG132, a proteasome inhibitor. WB analysis revealed that MG132 treatment restored c-Myc expression levels that had been reduced by *CENPF* knockdown, implying that CENPF modulates c-Myc stability via the ubiquitin-proteasome system (Fig. 7I,J). Finally, Co-immunoprecipitation experiments showed that MG132 treatment could rescue c-Myc protein levels in *CENPF*-knockdown cells, further confirming that CENPF regulates c-Myc protein stability through the proteasome pathway (Fig. 7K).

### 3.9 CENPF Enhances GC Cell Proliferation by Upregulating GLS1 Expression through c-Myc Activation

**Figure 8:**
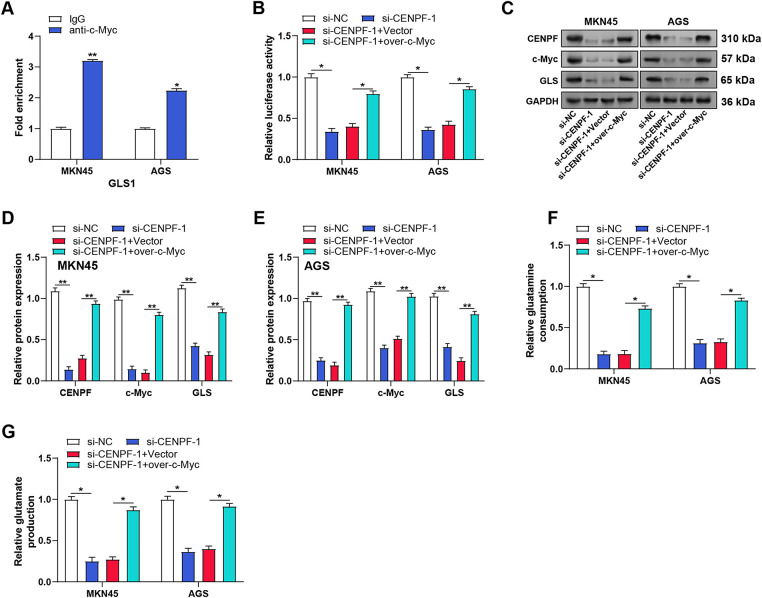
*CENPF* enhances *GLS* expression via *c-Myc* activation in GC cells. (**A**) ChIP-qPCR analysis of *c-Myc* binding to the *GLS* promoter in MKN45 and AGS cells. (**B**) Luciferase reporter assays showing GLS promoter activity in MKN45 and AGS cells with indicated treatments. (**C**–**E**) WB analysis of the effects of *CENPF* knockdown and c*-Myc* overexpression on c-Myc, GLS1, and CENPF protein levels in MKN45 and AGS cells. (**F**) Glutamine consumption assay showing the effects of *CENPF* knockdown and *c-Myc* overexpression on glutamine uptake in MKN45 and AGS cells. (**G**) Glutamate production assay demonstrating the influence of *CENPF* knockdown and *c-Myc* overexpression on glutamate generation in MKN45 and AGS cells. GC: gastric cancer; CHIP-qPCR: chromatin immunoprecipitation followed by quantitative polymerase chain reaction. *p < 0.05 vs. IgG or si-NC or si-*CENPF*-1 + Vector, **p < 0.01 vs. IgG or si-NC or si-*CENPF*-1 + Vector

**Figure 9:**
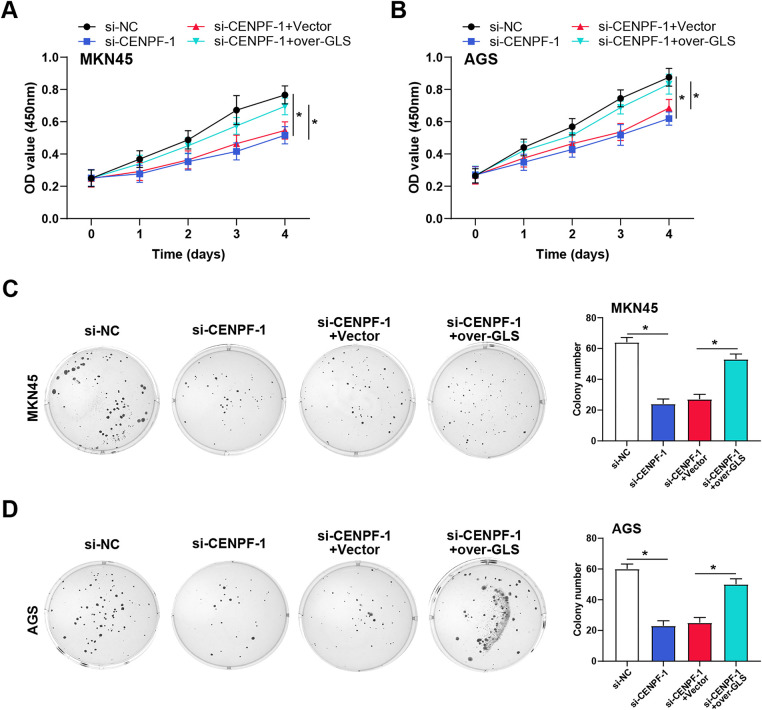
*CENPF* promotes GC cell proliferation by regulating GLS1 expression. (**A**,**B**) CCK-8 assays were performed to assess the effect of *CENPF* knockdown and *GLS* overexpression on cell viability in MKN45 (**A**) and AGS (**B**) cells. (**C**,**D**) Colony formation assays were conducted to evaluate the impact of *CENPF* knockdown and *GLS* overexpression on cell proliferation in MKN45 (**C**) and AGS (**D**) cells. Images are shown on the left, with corresponding bar graphs representing the cell number on the right. GC: gastric cancer; CCK-8: cell counting kit-8. *p < 0.05 vs. si-NC or si-*CENPF*-1 + Vector

The literature by Yang et al. provided potential *c-Myc* binding sites in the *GLS1* promoter region [22]. The direct binding of *c-Myc* to the *GLS* promoter in AGS and MKN45 cells was validated by ChIP-qPCR experiments (Fig. 8A). Luciferase reporter assays showed that *CENPF* knockdown decreased *GLS* promoter activity, while *c-Myc* overexpression rescued this effect (Fig. 8B), suggesting a potential interaction between *CENPF* and *c-Myc* in regulating gene expression. WB analysis of c-Myc, GLS1, and *CENPF* protein expression in MKN45 and AGS cells confirmed that *CENPF* knockdown suppressed the expression of all three proteins, while *c-Myc* overexpression reversed the effects of *CENPF* knockdown (Fig. 8C–E). Furthermore, metabolic analysis revealed that the decreased glutamine consumption and glutamate production caused by *CENPF* knockdown could be reversed by *c-Myc* overexpression (Fig. 8F,G). To verify the functional relationship between *CENPF* and *GLS*, we conducted rescue trials in MKN45 and AGS cells. CCK-8 assays showed that *CENPF* knockdown suppressed cell proliferation, while *GLS* overexpression partially restored the proliferation capacity of both cell lines (Fig. 9A,B). Consistent with these findings, colony formation assays showed that the decreased colony formation ability caused by *CENPF* knockdown could be effectively rescued by *GLS* overexpression in MKN45 and AGS cells (Fig. 9C,D). These results suggest that *CENPF* promotes GC cell proliferation by upregulating *GLS* expression through *c-Myc* activation, highlighting a critical role in glutamine metabolism.

## 4 Discussion

As a widely prevalent malignant tumor, GC continues to provoke extensive research aimed at uncovering its underlying pathogenesis and identifying effective therapeutic strategies. In this study, differential expression analysis and PPI network analysis were employed to identify ten genes closely associated with GC, including *FOXM1*, *NUF2*, *SPAG5*, and others. Tang et al. demonstrated that *FOXM1* enhances the stability of hTERT protein through a non-transcriptional mechanism, which is linked to the poor prognosis and progression of GC [23]. Long et al. showed that a poor prognosis is linked to considerably higher *NUF2* expression in GC [24]. *NUF2* promotes GC progression by regulating the G2/M transition and inhibiting apoptosis. Furthermore, the *NUF2* inhibitor quercetin exhibited potent anti-cancer effects in GC cells and models. A study has found that *SPAG5* is linked to a negative outcome and is significantly expressed in GC [25]. Moreover, silencing *SPAG5* can inhibit GC cell proliferation, induce apoptosis, and enhance sensitivity to 5-fluorouracil. Furthermore, *SPAG5* promotes tumor growth by controlling the PI3K/AKT signaling cascade. In summary, the aberrant expression and functional relevance of these genes emphasize their involvement in GC development and highlight their potential clinical relevance.

*CENPF* is part of the centrin protein family and is primarily engaged in interactions with microtubules, spindle stability, and the regulation of the cell cycle. Xiao et al. revealed that *CENPF* is upregulated in endometrial carcinoma (EC) tissues and encourages EC progression by enhancing cell invasion, proliferation, migration, and accelerating the cell cycle [26]. Additionally, *CENPF* exerts its effects in EC cells by regulation of the PI3K/AKT/mTOR pathway. Xu et al. suggested that the m^6^A modification of *CENPF* is regulated by methyltransferase 3, which enhances its mRNA stability through *HNRNPA2B1*, thereby promoting the role of *CENPF* in GC [27]. *CENPF* facilitates GC metastasis by epithelial-to-mesenchymal transition and the CENPF/FAK/MAPK axis, and its high expression is correlated with clinical features in GC patients. Zhao et al. reported that the serum expression levels of *CENPF* are elevated in advanced gastric cancer patients, while the levels of miR-1-3p are significantly decreased [28]. Both of these factors are associated with prognosis and may serve as risk indicators for GC prognosis. Consistently, our additional analysis of the TCGA-STAD dataset and recent literature evidence indicates that high *CENPF* expression correlates with poorer overall survival and adverse clinicopathological characteristics not only in gastric cancer but also in other malignancies such as hepatocellular carcinoma, lung cancer, and thyroid carcinoma [29–31]. These findings further support the prognostic value of *CENPF* across multiple cancer types and reinforce its potential as a biomarker for patient stratification. Consistent with these results, our study showed that *CENPF* was significantly upregulated in GC cell lines compared with normal gastric epithelial cells, and knockdown of *CENPF* in GC cells resulted in a significant decrease in their proliferation capacity. These data highlight the pro-proliferative role of *CENPF* in gastric cancer and suggest its involvement in the maintenance of the malignant phenotype. In addition, after knockdown of *CENPF*, the apoptosis rate increased, and cells in the G2 phase aggregated. The downregulated expression of key regulatory factors such as c-Myc, Cyclin B1, and CDK1 further confirmed that *CENPF* promotes GC progression by promoting cell cycle progression and inhibiting apoptotic signaling. These results point to *CENPF*’s critical carcinogenic function in GC progression, but the specific molecular mechanisms remain to be elucidated.

The reliance on glutamine by tumor cells, often referred to as “glutamine addiction” is a well-established phenomenon wherein tumor cells take up large amounts of glutamine through highly expressed *SLC1A5* transporters to support rapid proliferation [32]. Within tumor cells, glutamine is catalyzed by GLS to produce glutamate, which then participates in the tricarboxylic acid (TCA) cycle and the synthesis of GSH [33]. Glutamine and its derivatives (such as glutamate) are elevated in gastric cancer tissues and can be used as markers for early diagnosis or efficacy monitoring. Liu et al. found that *RUNX3* can inhibit glutamine metabolism by upregulating circDYRK1A in GC cells, thereby inhibiting cancer progression [34]. In this context, this study performed RNA sequencing and enrichment analysis on GC cells with knockdown of *CENPF*, revealing a potential link between *CENPF* and glutamine metabolism. GO-BP and KEGG analyses showed that DEGs were significantly enriched in pathways related to the biosynthesis and transport of amino acids, while GSEA analysis showed that there was a close association between *CENPF* and alanine, aspartate, and glutamate metabolic pathways. This pathway is a key branch of amino acid metabolism, intersecting with TCA and regulating cell survival under metabolic stress [35]. This means that *CENPF* may be involved in controlling glutamine addiction in GC, further emphasizing the possibility of intervening in glutamine metabolic targets. Our experimental results further confirmed these mechanisms. *CENPF* overexpression promoted glutamine metabolism and conferred cell resistance to apoptosis, while these effects were abolished under glutamine-deficient conditions. These results suggest that *CENPF* promotes GC cell survival by maintaining glutamine-driven metabolic flux.

GLS is the critical enzyme responsible for converting glutamine into glutamate, and its upregulation typically enhances glutamine metabolism in cancer cells, supporting their proliferation and survival [36]. Yu et al. comprehensively reviewed glutamate metabolism and its significance in cancer, particularly focusing on the “kidney-type” GLS1, which is commonly overexpressed in rapidly proliferating cancer cells [37]. Inhibition of *GLS1* was proposed as a possible cancer treatment method. Li et al. summarized the processes of glutamine absorption, transport, and metabolism in tumor cells, highlighting its central function in regulating non-essential amino acids (NEAAs), fatty acids, nucleotides, and ATP synthesis [32]. CB-839 is a GLS inhibitor targeting glutamine metabolism. It blocks the conversion of glutamine to glutamate by inhibiting *GLS1*, thereby interfering with the energy metabolism and biosynthesis of tumor cells. In lung adenocarcinoma, CB-839 combined with the polyamine metabolism drug DENSpm synergistically inhibited tumor growth and enhanced efficacy by blocking glutamine metabolism and GSH synthesis [38]. Our study showed that *GLS* overexpression restored the proliferation capacity and inhibited apoptosis of *CENPF*-silenced GC cells, suggesting that *GLS* is a key downstream effector of *CENPF*. Conversely, silencing *GLS* reversed the growth-promoting and anti-apoptotic effects of *CENPF* overexpression, indicating that the role of *CENPF* in glutamine metabolism is largely dependent on *GLS*. This regulatory axis was further validated by using CB-839, an inhibitor that abolished *CENPF*-mediated enhancement of cell viability and glutamate/α-KG production while promoting apoptosis. These findings suggest that *CENPF* may modulate glutamine metabolism in GC cells, reinforcing its potential function in cancer progression and offering new perspectives for targeted therapies.

*c-Myc* acts as a transcriptional regulator that promotes rapid cancer cell growth by regulating various metabolic pathways, particularly those engaged in the metabolism of amino acids, fatty acids, and glucose [39,40]. Zhao et al. demonstrated that glutamine deprivation activates *c-Myc*, which in turn stimulates the expression of *GOT1*, maintains GSH synthesis, and inhibits ferroptosis [41]. *SMYD2* stabilizes c-Myc by methylation and has been shown to upregulate *GLS1* expression, promote glutamine metabolism, and thus enhance the resistance of liver cancer cells to sorafenib [42]. c-Myc-driven glutamine addiction has also been detected in bladder cancer, thereby promoting the malignant progression of cancer [43]. These studies suggest the possibility that *c-Myc* participates in metabolic reprogramming by regulating *GLS1*. We demonstrated that *CENPF* stabilizes c-Myc protein and prolongs its half-life by inhibiting ubiquitin-proteasome-mediated degradation. Our data reveal a positive regulatory axis in which *CENPF* promotes the stability of c-Myc, c-Myc transactivates *GLS1*, and the resulting upregulation of *GLS1* enhances glutamine decomposition, thereby promoting GC cell proliferation. Functional analysis further validated this regulatory axis. Knockdown of *CENPF* impaired c-Myc protein stability, downregulated *GLS1* expression, and inhibited GC cell proliferation and colony formation. Overexpression of *c-Myc* or *GLS1* reversed these effects, confirming the functional dependency of this pathway. These findings propose that the *CENPF*-*c-Myc*-*GLS* axis is critical to glutamine metabolism and GC progression, offering potential targets for therapeutic intervention. Recent studies have revealed that both *CENPF* and *c-Myc* are involved in regulating metabolic reprogramming across multiple cancer types. For instance, *CENPF* has been reported to promote proliferation and metastasis through cell-cycle and metabolic regulation in prostate and breast cancers [20,44–46]. Meanwhile, c-Myc serves as a master regulator of glutamine metabolism in various malignancies, including hepatocellular carcinoma and colorectal cancer, by upregulating *GLS1* and enhancing glutamine utilization [47,48]. Given these findings, the *CENPF/c-Myc/GLS* axis may represent a common regulatory mechanism underlying tumor growth and metabolic adaptation. Our study extends this concept to gastric cancer, revealing that *CENPF* enhances glutamine metabolism and tumor progression through the *c-Myc/GLS1* signaling cascade. These results not only underscore the critical role of metabolic reprogramming in gastric cancer but also suggest that targeting the *CENPF/c-Myc/GLS* axis could represent a novel and broadly applicable therapeutic strategy across multiple cancer types.

Recent studies have revealed that *CENPF* is involved in gastric cancer progression via multiple mechanisms. For example, hnRNPR stabilizes *CCNB1/CENPF* mRNA to promote GC cell proliferation and metastasis [14], with such studies highlighting *CENPF*’s role in cell cycle regulation. Our findings expand this understanding by linking CENPF to metabolic regulation, particularly glutamine metabolism, via the *CENPF-c-Myc-GLS1* axis. Unlike the hnRNPR-CCNB1/CENPF pathway, which primarily affects cell cycle progression, our study demonstrates that *CENPF* overexpression enhances glutamine consumption and glutamate production through *c-Myc*-mediated transcriptional activation of *GLS*, influencing both proliferation and apoptosis in GC cells. These complementary mechanisms suggest that *CENPF* exerts multifaceted oncogenic effects, integrating cell cycle control and metabolic reprogramming. As highlighted in *Overview of perspectives on cancer*, *newer therapies*, *and future directions* [49], cancer treatment is shifting toward personalized strategies targeting tumor-specific molecular mechanisms and the tumor immune microenvironment (TIME)—a trend that aligns with our findings on *CENPF*. The *CENPF-c-Myc-GLS1* axis we identified represents a key metabolic vulnerability in GC, especially for patients with high *CENPF* expression (validated in TCGA-STAD/GEO datasets). Notably, several natural compounds can modulate tumor glutamine metabolism and may offer new avenues to target this axis. Inhibition of *c-Myc* reduces glutaminolysis and tumor growth [50], while xanthohumol, berberine, and epigallocatechin-3-gallate (EGCG) suppress glutamine uptake or synergize with GLS inhibitors to enhance antitumor efficacy [51–53]. Given that *CENPF* stabilizes c-Myc and promotes *GLS1*-mediated glutamine metabolism, these findings suggest that natural products targeting *c-Myc* or *GLS* could indirectly attenuate *CENPF*-driven metabolic reprogramming. Although further experimental validation is needed, natural-product-based modulation of the *CENPF–c-Myc–GLS* axis represents a potential strategy for metabolic therapy in gastric cancer.

Clinically, targeting the *CENPF-c-Myc-GLS1* axis may have translational potential. For instance, the GLS inhibitor CB-839 [54], currently under clinical investigation, could be combined with strategies to inhibit *CENPF* or *c-Myc* [22] to suppress glutamine metabolism and tumor growth synergistically. Additionally, *CENPF*’s correlation with *c-Myc* (a known regulator of PD-L1) in our TCGA analysis hints at crosstalk with the TIME; combining *CENPF* inhibitors with immune checkpoint inhibitors (ICIs) may dualistically target metabolic dysregulation and immune suppression, benefiting *CENPF*-high, ICI-resistant GC patients. This approach not only bridges insights from *CENPF’s* cell cycle regulatory role and newly identified metabolic function but also aligns with the precision therapy paradigm emphasized in *Overview of perspectives on cancer*, *newer therapies*, *and future directions* [49], offering a tailored, effective direction for GC treatment.

Beyond therapeutic targeting, the clinical utility of *CENPF* extends to potential applications in non-invasive disease monitoring, a critical frontier in personalized cancer management. Liquid biopsy enables non-invasive cancer monitoring, with EVs and ctDNA emerging as key biomarkers. As highlighted in *Updates on liquid biopsies in neuroblastoma for treatment response*, *relapse and recurrence assessment*, *2024* [55], such tools support longitudinal tracking and personalized therapy. Our study identifies *CENPF* as a GC marker with high diagnostic accuracy (AUC = 0.839 in GSE19826, AUC = 0.823 in GSE27342). If *CENPF* is detectable in GC patients’ serum EVs or ctDNA, it could serve dual roles: (1) non-invasive early detection (especially in high-risk populations); (2) longitudinal monitoring of *CENPF-c-Myc-GLS* axis activation to guide therapy (e.g., CB-839 response). Unlike GLS1 (influenced by non-tumor factors), *CENPF* is GC-specific and correlates with tumor stage, reducing false positives. Future work will validate *CENPF* in clinical liquid samples to develop actionable GC biopsy panels.

Several limitations should be noted. First, due to limited clinical data, the association between *CENPF* expression and chemotherapy resistance in gastric cancer patients was not analyzed. Second, mechanistic experiments were performed in a limited number of cell lines, which may restrict the generalizability of our findings. Third, although our results suggest that CENPF stabilizes c-Myc via the proteasome, direct evidence for c-Myc ubiquitination regulation and physical interactions with *CENPF* is lacking. Finally, the linear presentation of results may overlook potential complexities or negative findings, highlighting the need for further validation in additional cell lines and *in vivo* models.

## 5 Conclusion

In conclusion, our study systematically identified and characterized *CENPF* as a pivotal oncogenic driver in GC, highlighting its function in promoting cell proliferation, inhibiting apoptosis, and regulating glutamine metabolism. Through comprehensive analysis, we demonstrated that *CENPF* upregulation was closely associated with key metabolic pathways, including the citric acid cycle and glutamine metabolism, which are essential for GC cell survival and proliferation. *In vitro* experiments confirmed that *CENPF* was markedly elevated in GC tissues and cell lines and encouraged cell proliferation and survival. *CENPF* knockdown induced G2 arrest and reduced the levels of key cell cycle regulators, suggesting a role in cell cycle progression. Mechanistically, *CENPF* stabilized c-Myc protein by preventing proteasomal degradation, which in turn directly bound to the *GLS* promoter and enhanced its transcription, thus establishing a regulatory axis connecting *CENPF*, *GLS*, and *c-Myc* in metabolic reprogramming, ultimately leading to increased glutamine metabolism in gastric cancer cells. These outcomes emphasize the prospects of *CENPF* as a therapeutic target for GC and provide new insights into the metabolic reprogramming of cancer cell proliferation. Future studies should focus on developing strategies to disrupt *CENPF*-mediated pathways, which may provide new avenues for targeted therapies for gastric cancer treatment.

## Data Availability

The datasets used and/or analyzed during the current study are available from the corresponding author upon reasonable request.
